# A Rare Case of Intracranial Hemorrhage Mimicking a Neoplasm in a Child With Hereditary Hemorrhagic Telangiectasia

**DOI:** 10.7759/cureus.87340

**Published:** 2025-07-05

**Authors:** Cristian Solano, Gleidson Silva, Thomas R VerHage, Tushar Chandra, Manish Bajaj

**Affiliations:** 1 Pediatric Radiology, Nemours Children's Hospital, Orlando, USA

**Keywords:** arteriovenous malformation, hereditary hemorrhagic telangiectasia (hht), neuroradiology, osler-weber-rendu syndrome, pediatric radiology, vascular malformations

## Abstract

Hereditary hemorrhagic telangiectasia (HHT) is an autosomal dominant disorder characterized by abnormal blood vessel formation, resulting in the development of arteriovenous malformations and telangiectasias throughout the body. We present a rare case of an intracranial hemorrhage mimicking a neoplasm in a medically complex pediatric patient with HHT, a diagnosis that was unbeknownst to both the clinical team and the interpreting radiologist early in the patient’s clinical course. This case not only demonstrates the importance of obtaining a thorough medical history but having a clear understanding of the numerous manifestations of HHT, especially as it pertains to the pediatric population. We implore strictly adhere to the guidelines of brain imaging at the time of diagnosis, as well as a multidisciplinary approach to the surveillance and management of these patients in hopes of preventing serious and potentially fatal complications.

## Introduction

Hereditary hemorrhagic telangiectasia (HHT), also known as Osler-Weber-Rendu syndrome, is an autosomal dominant disorder characterized by abnormal blood vessel formation, resulting in the development of arteriovenous malformations (AVMs) and telangiectasias throughout the body. Within the United States, the estimated prevalence of HHT has been proposed to be approximately 12.1 per 100,000 persons [1]. The clinical diagnosis of HHT is based on the presence of epistaxis, telangiectasias, visceral lesions, and family history as based on the established and internationally accepted Curaçao criteria [2-4]. Furthermore, the development of genetic studies has provided additional diagnostic tools to clinicians [5]. However, imaging remains an essential tool for both the diagnosis and identification of the numerous complications related to HHT, including, but not limited to, gastrointestinal, pulmonary, and intracranial hemorrhage. Unfortunately, these detrimental events may be the presenting symptom of HHT, especially in the pediatric population [6]. Therefore, if a pediatric patient is undergoing imaging evaluation and is suspected to have HHT, conveying that clinical concern to the interpreting radiologist is vital. A more focused search pattern in these cases may both reduce misidentification and increase the sensitivity of finding the numerous imaging sequelae of HHT, including the serious complication of hemorrhage.

## Case presentation

**Figure 1:**
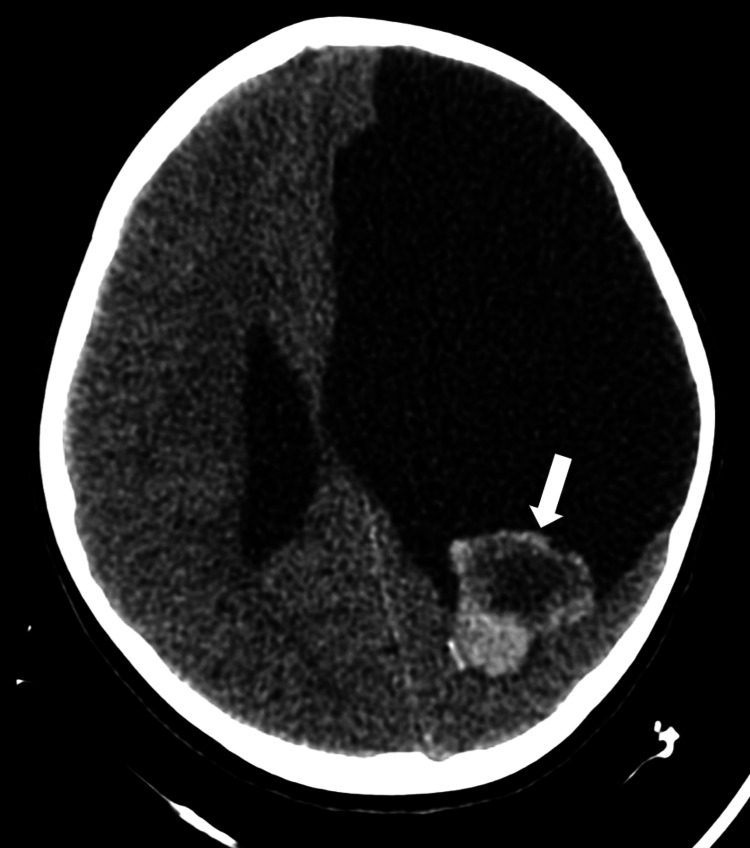
Initial axial non-contrast head CT. A heterogeneous hyperdense lesion measuring 3.1 x 2.9 x 3.3 cm was identified (arrow).

This is a case of a six-year-old medically complex pediatric female patient with a pertinent medical history of intrauterine stroke with residual right-sided spastic hemiparesis and epilepsy. Approximately one week before transfer to our institution, the patient presented to an outside emergency department (ED) after experiencing an isolated one-minute non-tonic-clonic breakthrough seizure. The patient was compliant with her anti-epileptic therapy prior to the event. This was the first seizure the patient had experienced since the age of eight months. Prior to the seizure, the patient endorsed a cough with posttussive emesis, congestion, and persistent headaches. A non-contrast CT head obtained at the outside ED reported a previously known large left-sided porencephalic cyst, as well as interval development of a 3.1 x 2.9 x 3.3 cm mass within the posterior aspect of the porencephalic cyst, postulated to be likely originating from the choroid plexus (Figure 1). The primary differential consideration offered at the time was a choroid plexus neoplasm, such as a papilloma. No acute intracranial abnormality was reported. A correlation of these findings with MRI was recommended.

**Figure 2:**
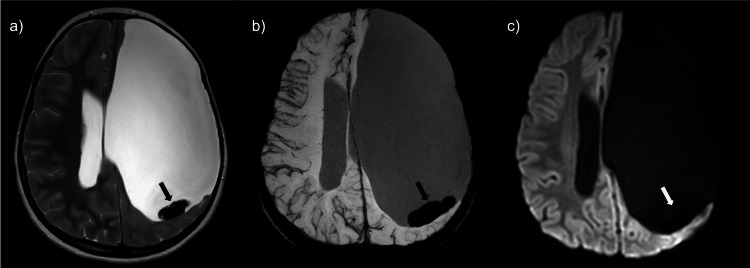
MR brain images demonstrate a significant decrease in the size of the lesion when compared to the prior CT. (a) Axial T2WI demonstrates a dependent T2 hypointense lesion (black arrow). (b) SWI shows blooming artifact consistent with blood products (black arrow). (c) DWI demonstrates no diffusion restriction (white arrow).

**Figure 3:**
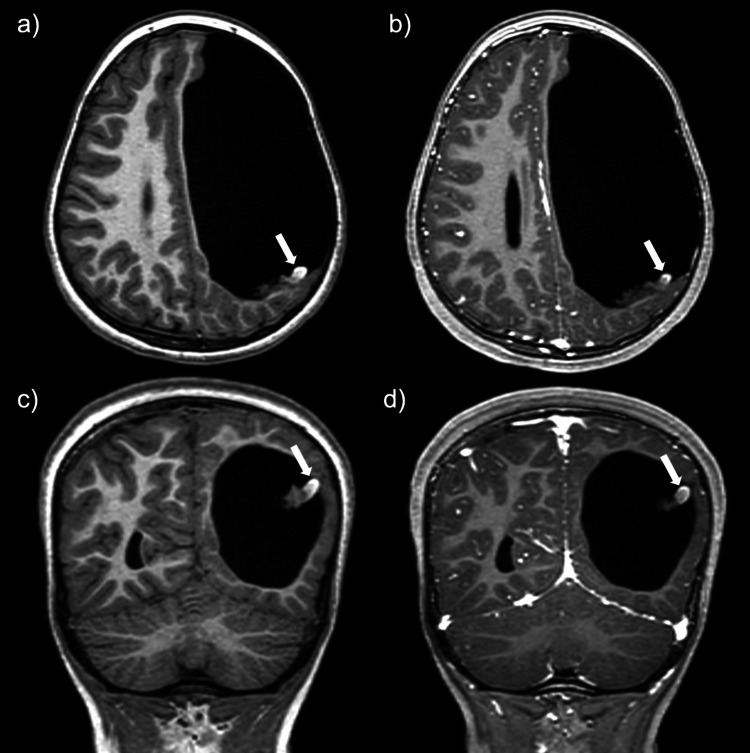
Pre- and post-contrast T1W brain images. Axial T1W pre-contrast (a) and post-contrast (b) and coronal T1W pre-contrast (c) and post-contrast (d) images show an inherently T1 hyperintense focus in the lateral aspect of the lesion (arrows) without post-contrast enhancement.

The patient returned to the ED and was eventually transferred to our facility for further workup and management. Upon arrival, an EEG showed no focal seizure activity. The patient was placed on a combination of dexamethasone, ketorolac, and acetaminophen for symptomatic management of the persistent headaches with mild initial improvement. An MRI brain with and without contrast, time-resolved magnetic resonance angiography (MRA), and magnetic resonance venography (MRV) were performed. MR brain images again demonstrated a large left-sided porencephalic cyst; however, there was a significant decrease in size of the previously reported lesion within the posterior aspect of the porencephalic cavity (previously perceived to represent a choroid plexus papilloma) (Figure 2). The lesion now measured 2.5 x 0.5 cm (previously 3.1 x 2.9 cm). This structure demonstrated predominantly low signal on T2-weighted imaging, with a focus of intrinsic high signal on T1-weighted imaging along the lateral margin of the lesion. Susceptibility-weighted images demonstrated diffuse and profound signal loss, most consistent with blood products. No contrast enhancement was identified (Figure 3).

**Figure 4:**
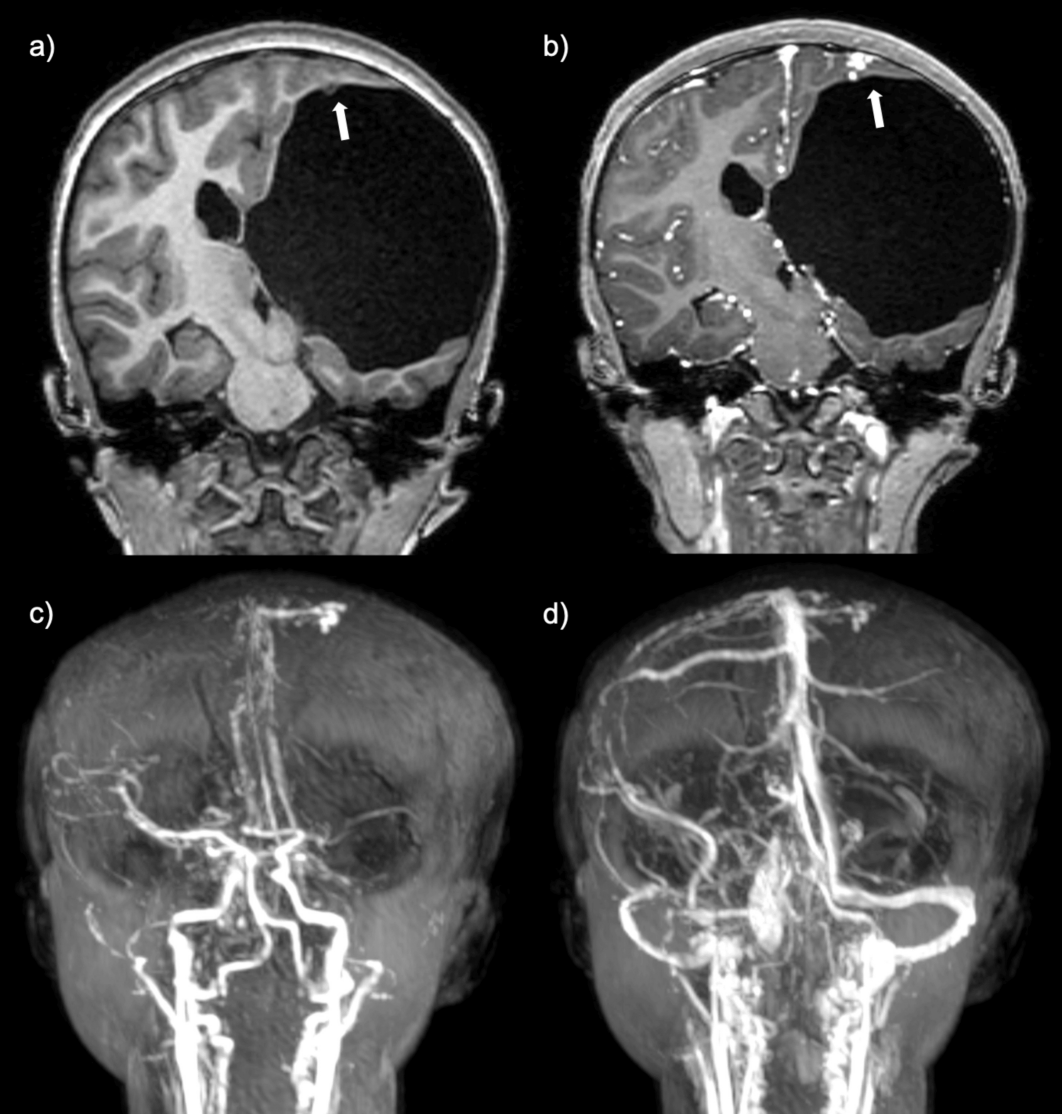
Coronal MR and MRA images. Coronal T1W pre-contrast (a) and post-contrast (b) images show a 0.8 x 0.6 cm tangle of vessels at the left high frontoparietal lobe with a subependymal component protruding into the superior aspect of the porencephalic cystic space. Time-resolved magnetic resonance angiography (MRA) MIP images demonstrate early arterial phase filling (c) with subsequent venous drainage (d) into a small cortical vein and then into the superior sagittal sinus, favored to represent a small arteriovenous malformation.

**Figure 5:**
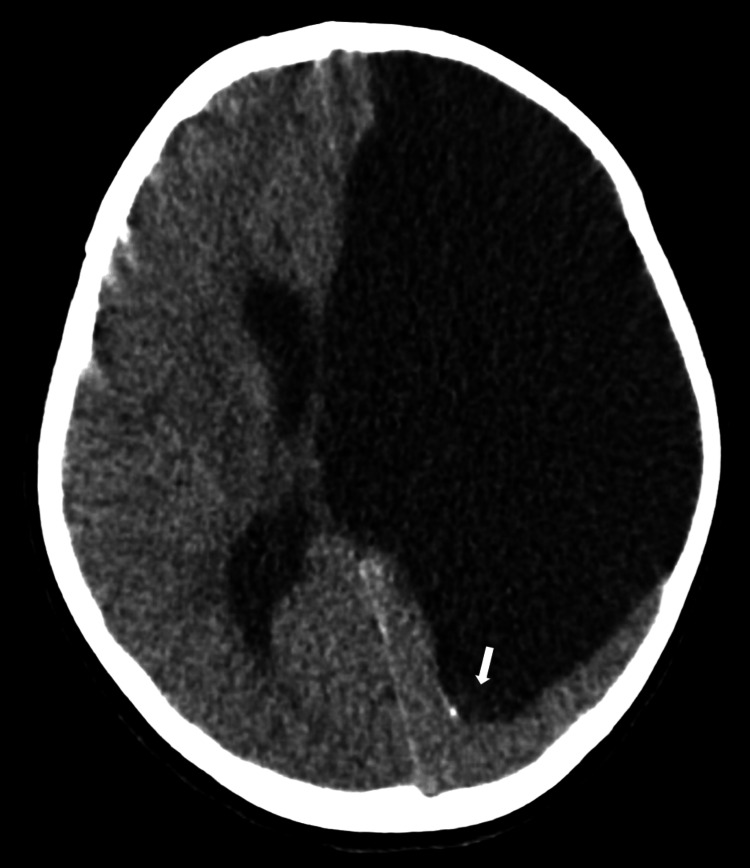
Axial non-contrast CT head nine days after the initial presenting CT head. The image shows near complete resolution of the dependent hematoma (arrow).

These findings were most suggestive of an evolving hematoma rather than an intracranial neoplasm as previously believed. Additionally, along the left high frontoparietal region, there was a 0.8 x 0.6 cm tangle of vessels with a subependymal component protruding into the superior aspect of the porencephalic cystic space. Time-of-flight MRA and MRV images demonstrated early arterial phase filling of the lesion, followed by venous drainage into a small cortical vein, which subsequently drained into the superior sagittal sinus, favored to represent a small arteriovenous malformation (Figure 4). Due to these findings, a more detailed evaluation of the patient’s medical history was performed, and it was elucidated that the patient and a first-degree relative were genetically diagnosed with hereditary hemorrhagic telangiectasia. This was unbeknownst to both the primary team and the radiologist early in the hospital course. Neurosurgery evaluated the patient and proposed that the persistent headache was likely secondary to meningeal irritation from subarachnoid hemorrhage, as well as a component of increased intracranial pressure from communicating hydrocephalus. After the MRI findings were reported, the patient underwent a lumbar puncture, which demonstrated an elevated opening pressure of 33 cm H_2_O (normal: 7-17 cm H_2_O) and yielded light pink-yellow cerebrospinal fluid (CSF). After the removal of approximately 15 mL of CSF, the closing pressure was measured to be 15 cm H_2_O. This briefly alleviated the patient's headache; however, the following day, the patient experienced a more severe headache and mild meningismus. A repeat CT head was performed and showed near complete radiographic resolution of the intracranial hematoma (Figure 5). However, given the worsening symptoms, a repeat LP was performed, which showed recurrent elevated opening pressures (34 cm H_2_O) with restoration of normal closing pressure after removal of 50 mL of CSF. As the symptoms persisted, the patient underwent placement of a left-sided lumboperitoneal shunt with a left frontal tapping reservoir. The remaining hospital course was unremarkable. The patient progressed well and was discharged shortly after surgery with instructions for outpatient clinical and imaging follow-up.

## Discussion

Over the past two decades, there has been a significant increase in the prevalence of HHT diagnosed in young adults (ages 18-29), a pattern which has not been observed in the pediatric population. This increase in prevalence in young adults has been attributed to the increased usage of the Curaçao criteria [1]. The Curaçao criteria for HHT pertains to the presence of the following: epistaxis (spontaneous and occurring more than once); telangiectasias (multiple at characteristic sites, such as nose, lips, oral cavity, and fingers); visceral lesions (AVMs at sites such as the central nervous system, lungs, liver and telangiectases of the GI tract); and family history (first degree relatives diagnosed with HHT based on the same criteria). HHT diagnosis is considered definite if three criteria are present, possible or suspected if two criteria are present, and unlikely if fewer than two criteria are present [2-4]. However, there is a slight limitation in the application of these criteria in young children, as the mean age of epistaxis onset is approximately 12 years of age in patients with HHT. In many children affected by HHT, this is well above the age of symptomatic onset, such as in the previously presented case [7]. As epistaxis is one of the four Curaçao diagnostic criteria, this may explain the prevalence discrepancy observed between children and young adults secondary to underdiagnosis. As such, a recent retrospective pediatric cohort study demonstrated that, if a patient meets only one criterion or two criteria (unlikely HHT or possible HHT), non-clinical criteria (i.e., genetic testing) are preferred for diagnosis. When a child or adolescent meets three or four criteria (definite HHT), the Curaçao criteria remain reliable [8]. This is especially important as delays in diagnosis may expose pediatric patients to the risks of the numerous unforeseen and potentially serious consequences related to HHT. If the child is diagnosed early, these risks could have either been addressed or, at the very least, monitored and prepared for.

It is this diagnostic dilemma that highlights the importance of not only a multidisciplinary approach in pediatric populations undergoing HHT evaluation but also exemplifies the role of non-clinical evidence to aid in the diagnosis and management of this condition. Given the serious complications associated with HHT, including gastrointestinal, pulmonary, and cerebral hemorrhage, early diagnosis, surveillance, and appropriate treatment can be essential in the clinical course of a pediatric patient [1]. Specifically, cerebral vascular malformations and the potential for intracranial bleeding can both be acutely devastating and have lasting clinical consequences in a child. A recent study by Kilian et al. [6] of 367 patients compared the characteristics of brain vascular malformations in children with HHT to their adult counterparts. This study demonstrated that a significantly smaller proportion of pediatric patients with brain venous malformations were diagnosed by conventional screening. Furthermore, a disproportionately large fraction of the pediatric cohort was symptomatic at presentation and experienced symptoms, such as headache, seizures, or other focal neurological deficits, likely related to the underlying venous malformations. Of note, headache was the most common presenting symptom in both adult and pediatric patients, a symptom noted in our patient at ED presentation. Finally, when compared to the adult cohort, a much greater degree (nearly a fourth) of the studied pediatric cohort presented with intracranial hemorrhage at the time of presentation [6].

Per the expert panel recommendations of the Second International Guidelines for the Diagnosis and Management of HHT, screening for brain venous malformations in asymptomatic children with HHT or at risk for HHT at the time of presentation or diagnosis is advised. The first line screening modality is with MRI brain imaging, preferably with and without contrast [4]. Radiology-specific guidelines recommend brain screening imaging with MRI with 3.0 Tesla post-contrast high-spatial-resolution with susceptibility-weighted imaging and time-of-flight MRA when possible [9]. Additionally, the expert panel recommends treatment of brain venous malformations with high-risk features; however, the high-risk features were not explicitly outlined [4]. Furthermore, universally accepted imaging surveillance guidelines have not been established at this time.

Of note, traditional high-risk features of venous malformation may differ in adults and children. A study published in 2012 found that smaller AVM size (defined as less than 3 cm), exclusive deep venous drainage, and an infratentorial location were specific and independently associated with initial hemorrhagic presentation in children with AVMs [10]. When compared to adults, high-risk features, such as venous ectasia and feeding artery aneurysms, were less commonly present in pediatric AVM cases. It may be possible that these features take time to develop [11].

Genetic studies are extremely valuable in the diagnosis of HHT, most notably in indeterminate cases [8]. Numerous gene mutations related to HHT have been identified within the transforming growth factor-beta signaling pathway, which is involved in a variety of essential cellular functions. The most commonly identified gene mutations involve endoglin (ENG), ACVRL-1, and SMAD-4, with the former two implicated in as much as 85% of cases [5,12]. Although there are no defined imaging surveillance guidelines, patients with known ENG mutations have been shown to be at an increased risk for cerebral AVM development than patients with the other well-known ACVRL-1 and SMAD-4 mutations [12]. As a result, these patients may benefit from closer imaging surveillance.

Although beyond the scope of this discussion, brain AVM treatment depends on the patient’s presentation and institution standards, ranging from surgery with or without embolization for ruptured AVMs to imaging surveillance for unruptured brain AVMs [9]. We implore strictly adhere to the guidelines of brain imaging at the time of HHT diagnosis. Furthermore, if an intracranial venous malformation is identified, the patient should undergo a multidisciplinary evaluation to devise an appropriate care plan rather than the practice of watchful waiting employed in the past. Additionally, if a pediatric patient presents with the complaint of a headache and there is high clinical suspicion of HHT, we believe it is reasonable to perform MR brain imaging to exclude underlying arteriovenous malformation and/or intracranial hemorrhage.

## Conclusions

We present a case of a medically complex six-year-old child, with an initially unknown HHT status, who presented with a persistent headache and a breakthrough seizure. In the absence of the clinical history of HHT, an intracranial hematoma mimicked the appearance of a neoplasm and was reported as such on CT head images. In this case, early identification of the patient’s HHT status may have accelerated the medical decision-making to reduce both the length and distress of the patient’s clinical course. In addition, as the interpreting radiologist, it is important to both consider and provide a wide differential diagnosis to the clinical team. As a result, we propose not only strictly adhering to the established guidelines of baseline brain MR imaging at the time of HHT diagnosis but also obtaining a prompt MRI for equivocal intracranial lesions, especially when only radiographic imaging is available. The additional MR information allows for more definitive evaluation of these lesions, such as in the previously presented case. As with other complex medical syndromes, we advise a multidisciplinary approach and discussion with the patient's family for continued disease understanding, surveillance, and potential treatment.

## References

[1] Ferry AM, Wright AE, Baillargeon G, Kuo YF, Chaaban MR (2020). Epidemiology and trends of hereditary hemorrhagic telangiectasia in the United States. Am J Rhinol Allergy.

[2] Shovlin CL, Guttmacher AE, Buscarini E (2000). Diagnostic criteria for hereditary hemorrhagic telangiectasia (Rendu-Osler-Weber syndrome). Am J Med Genet.

[3] Faughnan ME, Palda VA, Garcia-Tsao G (2011). International guidelines for the diagnosis and management of hereditary haemorrhagic telangiectasia. J Med Genet.

[4] Faughnan ME, Mager JJ, Hetts SW (2020). Second international guidelines for the diagnosis and management of hereditary hemorrhagic telangiectasia. Ann Intern Med.

[5] McDonald J, Wooderchak-Donahue W, VanSant Webb C, Whitehead K, Stevenson DA, Bayrak-Toydemir P (2015). Hereditary hemorrhagic telangiectasia: genetics and molecular diagnostics in a new era. Front Genet.

[6] Kilian A, Latino GA, White AJ (2023). Comparing characteristics and treatment of brain vascular malformations in children and adults with HHT. J Clin Med.

[7] AAssar OS, Friedman CM, White RI Jr (1991). The natural history of epistaxis in hereditary hemorrhagic telangiectasia. Laryngoscope.

[8] Pahl KS, Choudhury A, Wusik K (2018). Applicability of the Curaçao criteria for the diagnosis of hereditary hemorrhagic telangiectasia in the pediatric population. J Pediatr.

[9] Hetts SW, Shieh JT, Ohliger MA, Conrad MB (2021). Hereditary hemorrhagic telangiectasia: the convergence of genotype, phenotype, and imaging in modern diagnosis and management of a multisystem disease. Radiology.

[10] Ellis MJ, Armstrong D, Vachhrajani S (2013). Angioarchitectural features associated with hemorrhagic presentation in pediatric cerebral arteriovenous malformations. J Neurointerv Surg.

[11] Hetts SW, Cooke DL, Nelson J (2014). Influence of patient age on angioarchitecture of brain arteriovenous malformations. AJNR Am J Neuroradiol.

[12] Azma R, Dmytriw AA, Biswas A (2022). Neurovascular manifestations in pediatric patients with hereditary haemorrhagic telangiectasia. Pediatr Neurol.

